# The Association between Maternal Endocrine-Disrupting Chemical Exposure during Pregnancy and the Incidence of Male Urogenital Defects: A Systematic Review and Meta-Analysis

**DOI:** 10.3390/metabo14090477

**Published:** 2024-08-29

**Authors:** Emad Ali Albadawi, Naweed SyedKhaleel Alzaman, Yasir Hassan Elhassan, Heba M. Eltahir, Mekky M. Abouzied, Muayad Saud Albadrani

**Affiliations:** 1 Department of Basic Medical Sciences, College of Medicine, Taibah University, Al-Madinah Al-Munawara 42354, Saudi Arabia; 2 Department of Medicine, College of Medicine, Taibah University, Al-Madinah Al-Munawara 42354, Saudi Arabia; 3 Department of Pharmacology and Toxicology, College of Pharmacy, Taibah University, Al-Madinah Al-Munawara 42354, Saudi Arabia; 4 Department of Biochemistry, Faculty of Pharmacy, Minia University, Minia 2431436, Egypt; 5 Department of Family and Community Medicine and Medical Education, College of Medicine, Taibah University, Al-Madinah Al-Munawara 42354, Saudi Arabia

**Keywords:** EDC, endocrine-disrupting chemicals, urogenital defects, hypospadias, cryptorchidism

## Abstract

The increasing incidence of hypospadias and cryptorchidism, coupled with the widespread presence of endocrine-disrupting chemicals (EDCs), has raised concerns about the potential impact of these environmental factors on male urogenital development. This systematic review and meta-analysis aims to evaluate the association between maternal exposure to various EDCs and the risk of hypospadias and cryptorchidism. We conducted a comprehensive search of PubMed, Scopus, Web of Science, and Cochrane databases from inception until May 2024. We included case-control and cohort studies that examined the association between maternal EDC exposure and hypospadias or cryptorchidism, reporting adjusted odds ratios (aOR) or crude odds ratios (cOR). Data were extracted and pooled using a random effects model, and heterogeneity was assessed using the Q test and I-square statistics. The risk of bias was evaluated using the Newcastle–Ottawa Scale (NOS). A total of 48 studies were included in the systematic review, with 46 studies included in the meta-analysis. The pooled analysis revealed a significant association between maternal EDC exposure and an increased risk of hypospadias (aOR = 1.26, 95% CI: 1.18–1.35, *p* < 0.0001) and cryptorchidism (aOR = 1.37, 95% CI: 1.19–1.57, *p* < 0.001). Subgroup analyses showed that exposure to pesticides, phthalates, alkyl phenolic compounds (ALKs), and heavy metals significantly increased the risk of hypospadias. In contrast, polychlorinated biphenyls (PCBs) did not show a significant association. Significant associations were found with pesticide and PCB exposure for cryptorchidism, but not with phthalate, ALK, or heavy metal exposure. Maternal exposure to certain EDCs is associated with an increased risk of hypospadias and cryptorchidism in male children. These findings underscore the importance of addressing environmental and occupational exposures during pregnancy to mitigate potential risks. Further research is needed to elucidate the mechanisms by which EDCs affect urogenital development and to develop effective interventions to reduce exposure among vulnerable populations.

## 1. Introduction

Hypospadias, the second most prevalent anomaly affecting male genitalia, is characterized by congenital hypoplasia of the ventral aspect of the penis, accompanied by displacement of the urethral outlet. It also manifests by disjunction of the corpus spongiosum, a foreskin hooding dorsally, and, occasionally, ventral chordee. Its pathophysiology is thought to be multifactorial, involving genetic and environmental causes [1,2]. Despite linking many gene mutations with hypospadias, most cases do not show any genetic alterations with notable functional implications [3,4,5]. Cryptorchidism, also identified as incomplete testicular descent, is a prevalent congenital genetic anomaly that could also be acquired [6]. Cryptorchidism is believed to affect approximately 8% of male newborns, with a well-known association with a risk of testicular cancer and reduced fertility later in life [7,8].

Owing to the increasing incidence of hypospadias and cryptorchidism in certain regions or periods, there is an elevated suspicion that the environmental chemicals, also known as endocrine disruptors, could play a crucial role as a potential risk factor for developing these male urogenital defects [9,10,11,12,13,14]. Since the 1940s, the capacity of certain synthetic chemicals to interfere with the human hormonal system has been evident, coinciding with the beginning of the utilization of diethylstilbestrol in preventing spontaneous abortions [15]. Nevertheless, the term “endocrine disruptor” remained undefined till 1991, at the Wingspread conference in Wisconsin [16]. During this conference, a group of specialists met to explore the underlying causes of health issues noted in individuals and animals in developed countries involving reproductive system disorders. As a result, an endocrine-disrupting chemical (EDC) was outlined as an exogenous agent that can disturb the hormonal balance, harming an organism or its offspring. The majority of these exogenous agents demonstrate estrogenic and antiandrogenic properties, potentially disrupting the androgen–estrogen balance during the developmental phase of a male fetus. EDCs also affect the process of external genital differentiation in both human and animal populations [17].

Interestingly, Sharpe and Skakkbaek suggested in 1993 that the elevated prevalence of reproductive system abnormalities noted in human males could result from increased estrogen exposure during intrauterine life [18]. Many widely prevalent chemicals involving dioxins, polychlorinated biphenyls (PCBs), pesticides, phthalate esters, and specific heavy metals have emerged as potential endocrine disruptors [19]. Pesticides, one of the common EDCs, were found to be associated with the development of congenital abnormalities if the child is exposed to them during the embryonic stage [20]. Parental involvement in agricultural work or pesticide exposure has been correlated with an elevated risk of congenital anomalies [21,22]. Moreover, Rocheleau et al. conducted a meta-analysis in 2009 to explore the association between pesticide exposure and hypospadias [23]. They concluded that pesticide exposure was associated with a high risk of hypospadias. Additionally, Yu et al. conducted a meta-analysis to examine the correlation between maternal phthalate exposure and male reproductive disorders, and they found that phthalate exposure was linked to a higher but not significant risk of developing hypospadias and cryptorchidism [24]. In our study, we delve into the intricate biomolecular mechanisms through which EDCs specifically impact urogenital development. These pathways involve hormonal signaling disruption, epigenetic modifications, and the delicate balance of hormones critical for fetal differentiation. While existing data gaps persist, we aim to bridge these knowledge deficits and contribute to a comprehensive understanding of EDC-related effects on urogenital health. Our meta-analysis investigates the association between maternal exposure to a wide range of EDCs and the risk of hypospadias and cryptorchidism. By comprehensively examining available data, we aim to enhance our understanding of these urogenital defects and contribute to informed preventive strategies.

## 2. Methods

To conduct this systematic review and meta-analysis study, the Cochrane Handbook rules were followed [25]. The PRISMA statement guidelines were followed during the reporting of this study [26].

### 2.1. Eligibility Criteria

We included all the studies that evaluated the association between maternal exposure to any EDC and hypospadias or cryptorchidism. The EDC includes pesticides with their relevant subtypes, alkyl phenolic (ALK) compounds, phthalates, heavy metals with their relevant subtypes, polychlorinated biphenyls (PCBs), and other EDCs. We only included case-control studies and cohort studies that assessed the relationship between EDC exposure and hypospadias or cryptorchidism as adjusted odds ratio (aOR) or crude odds ratio (cOR) or provided data to calculate them.

We excluded single-arm studies, studies that did not measure the association between EDC exposure and hypospadias or cryptorchidism or provide data to calculate it, conference abstracts, studies accessing the relationship between EDC and hypospadias or cryptorchidism as one outcome and did not separate them, review articles, and studies that included populations different from what we specified, as our study focuses on pregnant women only, specifically those who have been exposed to endocrine-disrupting chemicals (EDCs). Fathers and non-pregnant individuals are not part of our study population. Nevertheless, we explicitly considered conflicts of interest, such as industry funding or author consulting, as part of our exclusion criteria.

We exclusively included studies involving human subjects, ensuring that the meta-analysis is based on real-world human data and not on experimental models.

### 2.2. Literature Search and Screening

PubMed, Scopus, Web of Science, and Cochrane were accessed from inception till May 2024 with no restrictions on language or geographical locations. Our search strategy included a mixture of terms related to EDC, like pesticides, phthalates, heavy metals, hypospadias, and cryptorchidism. A detailed search strategy for each database is outlined in Supplementary Table S1.

Retrieved studies from the digital search were evaluated in a two-step screening process: first reviewing the title and abstracts for any eligible articles and then retrieving and reviewing the full text of eligible articles according to our specific criteria. Endnote (Clarivate Analytics, Philadelphia, PA, USA) was used to remove the duplicate articles.

### 2.3. Data Extraction

Data were collected using a pre-designed extraction sheet. We gathered the following data: a summary of the included articles, which includes study design, location, the total number of cases, the total number of controls, exposure assessment method, main chemicals, and conclusion.

### 2.4. Risk of Bias

Newcastle–Ottawa (NOS) was utilized to examine the risk of bias in the included studies [27]. NOS evaluates the risk of bias in observational studies according to three main domains: (1) selection of the study patients, (2) comparability between the study cohorts, and (3) exposure assessment. NOS ranks the studies according to calculated scores as good, moderate, or poor quality.

### 2.5. Statistical Analysis

OR and its corresponding 95% confidence interval were utilized to pool the association between EDC exposure and hypospadias and cryptorchidism. The random effect model was utilized to calculate the pooled ORs and their 95% confidence intervals, and the results were considered significant if the *p*-value was less than 0.05. Heterogeneity was assessed using the Q test and I-square. A *p*-value less than 0.1 or an I-square of more than 50% were considered significant heterogeneities. The primary analysis was done using two approaches: pooling the collected aOR and pooling the cOR. We also conducted a specific subgroup analysis based on the type of EDC and grouped them into pesticides, phthalates, heavy metals, PCBs, and other compounds. Suppose the study reported only EDC exposure as one chemical or reported other chemicals not related to any of the above subgroups. In that case, we put all these studies and their OR in the other subgroup. Publication bias was examined by the visual inspection of the funnel plot. The analysis was conducted using RevMan (V.5.3) for Windows.

## 3. Results

### 3.1. Search Results

**Figure 1:**
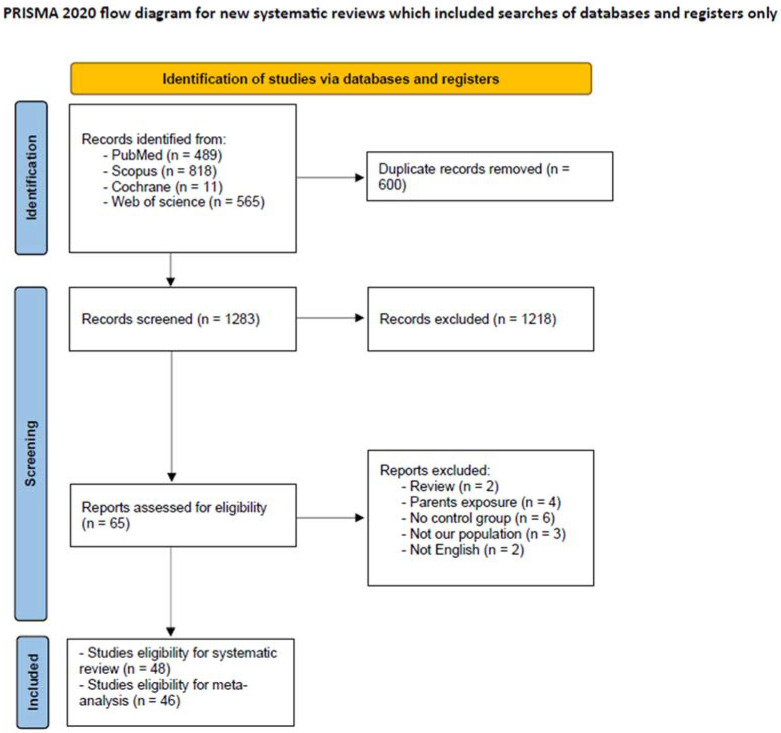
PRISMA Flow chart.

Our initial digital search retrieved 1883 studies, and 600 articles were removed as duplicates using Endnote. The results showed that 1283 articles were eligible for title and abstract screening. After the title and abstract screening, 1218 articles were excluded, and the full texts of 65 articles were retrieved for the second screening phase. Finally, 48 articles were involved in our study. Figure 1 outlines the PRISMA flow diagram of the study selection process.

### 3.2. Characteristics of the Included Studies

Forty-eight studies were included in our systematic review; of them, forty-six studies were included in the meta-analysis, with a total of 36,020 hypospadias cases, 27,960 cryptorchidism cases, and 790,277 controls [23,28,29,30,31,32,33,34,35,36,37,38,39,40,41,42,43,44,45,46,47,48,49,50,51,52,53,54,55,56,57,58,59,60,61,62,63,64,65,66,67,68,69,70,71,72,73,74,75]. All included studies were observational studies, and most of them were case-control studies. These studies were done in various countries, mainly in the USA (twelve studies), France (nine studies), Denmark (seven studies), and Spain (six studies). Detailed characteristics of the included studies are outlined in Table 1.

### 3.3. Quality Assessment

NOS was utilized to assess the quality of the included observational studies. Most of the included studies showed good quality, eight showed moderate quality, and only two showed poor quality. The detailed risk of bias for each study was presented in Supplementary Tables S2 and S3.

### 3.4. Study Outcomes

#### 3.4.1. Hypospadias

**Figure 2:**
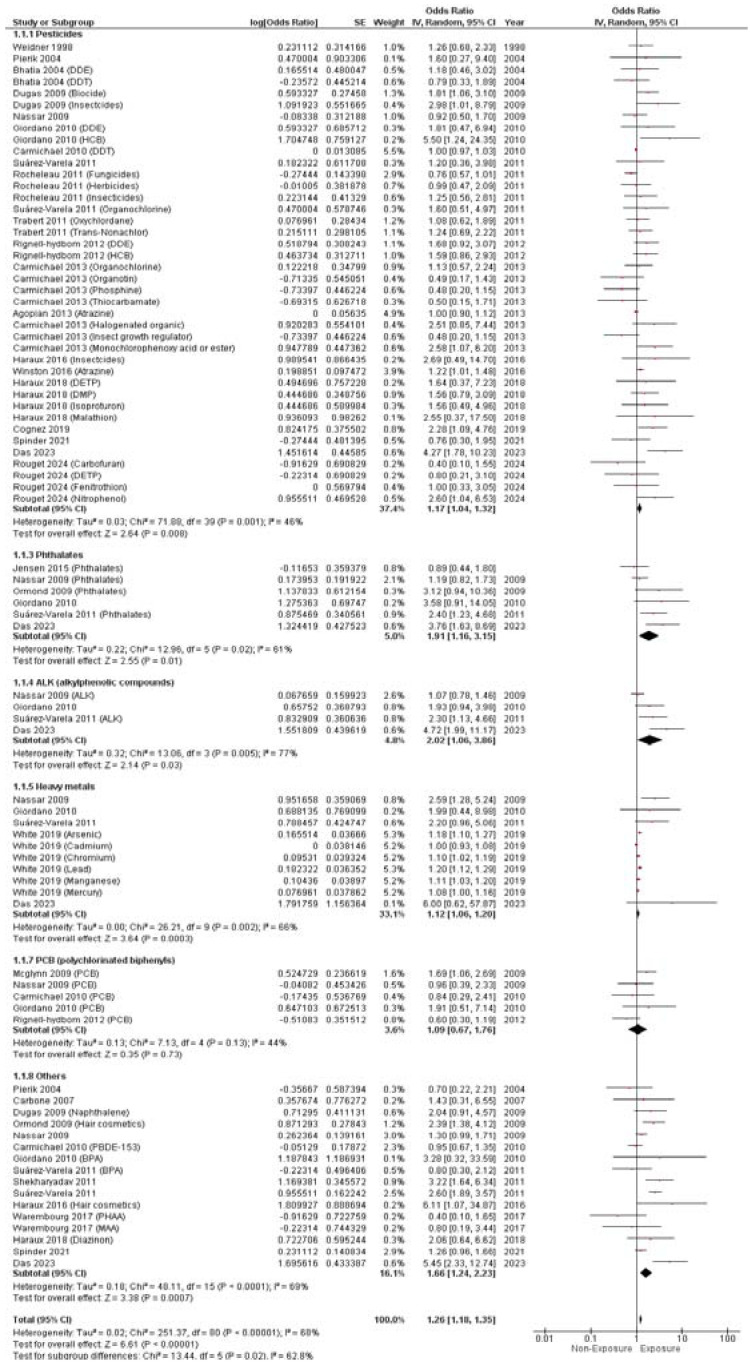
Forest plot of hypospadias incidence (adjusted OR).

The pooled aOR revealed that maternal EDC exposure was significantly associated with an elevated risk of hypospadias (aOR = 1.26, 95% CI (1.18,1.35), *p* < 0.0001). The pooled studies showed significant heterogeneity (I^2^ = 68%, *p* < 0.0001), as illustrated in Figure 2.

Additionally, pesticide, phthalate, ALK, and heavy metal exposure also showed an elevated risk of hypospadias (aOR = 1.17, 95% CI (1.04, 1.32), *p* = 0.008), (aOR = 1.91, 95% CI (1.16, 3.15), *p* = 0.01), (aOR = 2.02, 95% CI (1.06, 3.86), *p* = 0.03), (aOR = 1.12, 95% CI (1.06, 1.20), *p* = 0.0003), respectively. Interestingly, PCB exposure did not show any significant risk of hypospadias (aOR = 1.09, 95% CI (0.67, 1.76), as illustrated in Figure 2.

**Figure 3:**
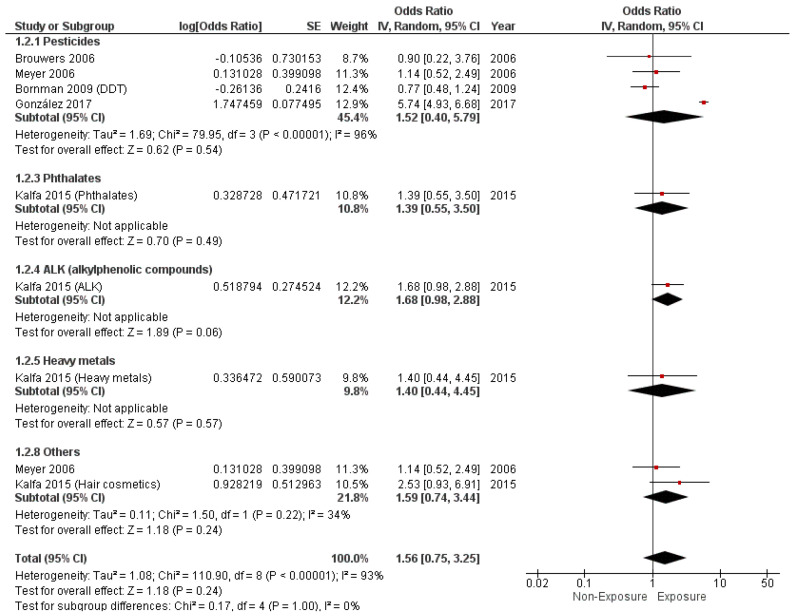
Forest plot of hypospadias incidence (crude OR).

The pooled cOR revealed no significant association between EDC exposure and hypospadias (cOR = 1.56, 95% CI (0.75, 3.25), *p* = 0.24). The pooled studies revealed significant heterogeneity (I^2^ = 93%, *p* < 0.0001). Moreover, pesticides (cOR= 1.52, 95% CI (0.40, 5.79), *p* = 0.54), phthalates (cOR= 1.39, 95% CI (0.55, 3.50), *p* = 0.49), ALKs (cOR = 1.68, 95% CI (0.98, 2.88), *p* = 0.06), and heavy metals (cOR = 1.40, 95% CI (0.44, 4.45), *p* = 0.57) also did not show any significant relationship with hypospadias, as illustrated in Figure 3.

**Figure 4:**
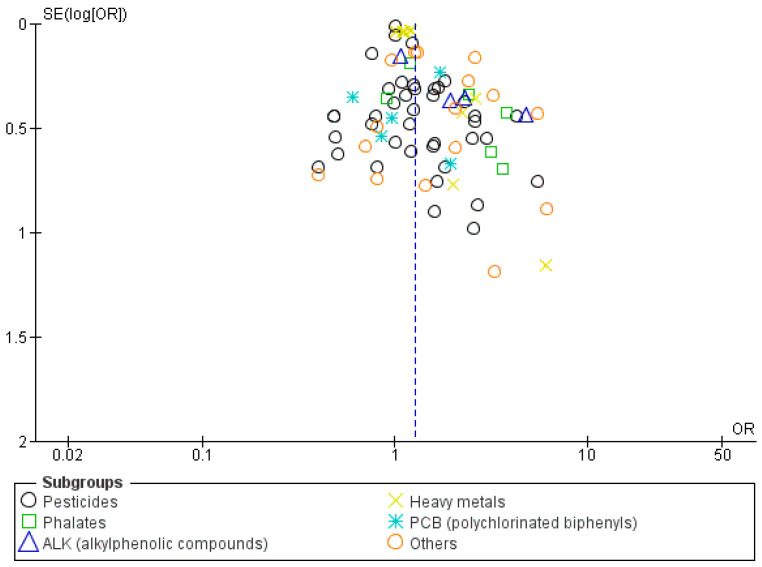
Funnel plot of hypospadias incidence (publication bias).

A funnel plot based on the adjusted odds ratio was constructed, and it showed asymmetry, indicating the possible presence of publication bias, as shown in Figure 4.

#### 3.4.2. Cryptorchidism

**Figure 5:**
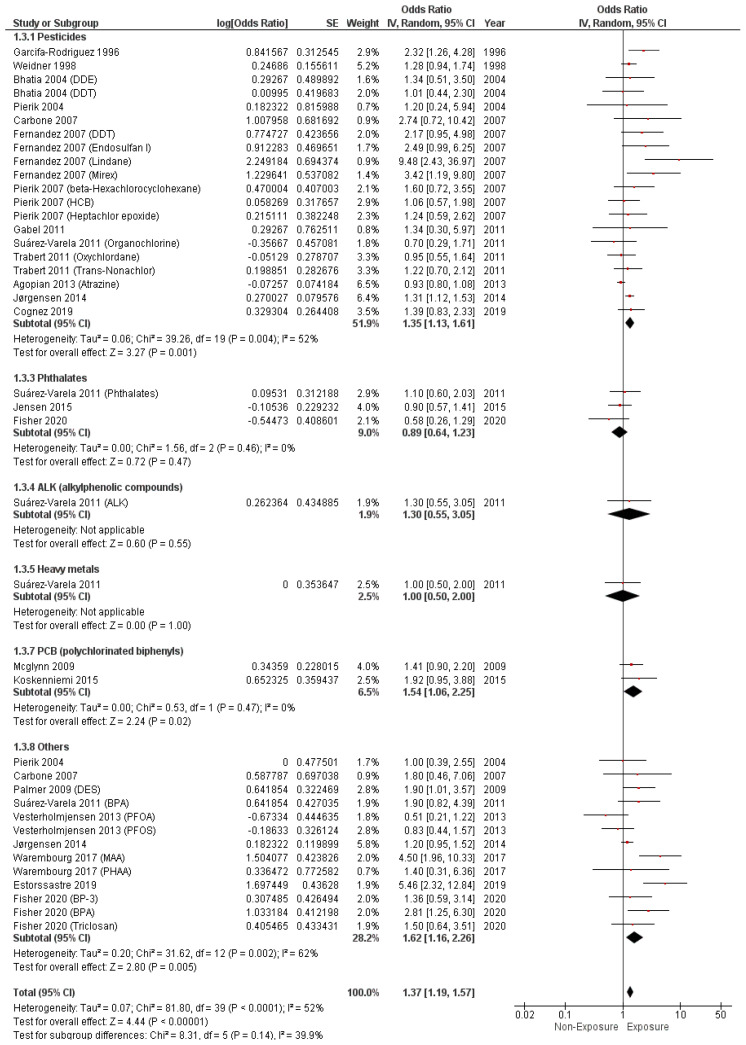
Forest plot of cryptorchidism incidence (adjusted OR).

The pooled aOR showed a significant association between maternal EDC exposure and cryptorchidism (aOR = 1.37, 95% CI (1.19, 1.57), *p* < 0.001). The pooled results demonstrated a significant heterogeneity (I^2^ = 52%, *p* < 0.0001), as illustrated in Figure 5.

Additionally, pesticide (aOR = 1.35, 95% CI (1.13, 1.61), *p* = 0.001) and PCB (aOR = 1.54, 95% CI (1.06, 2.25), *p* = 0.02) exposure were found to be associated with an increased risk of cryptorchidism.

Phthalate (aOR = 0.89, 95% CI (0.64, 1.23), *p* = 0.47), ALK (aOR = 1.30, 95% CI (0.55, 3.05), *p* = 0.55), and heavy metal (aOR = 1.00, 95% CI (0.50, 2.00), *p* = 1.00) exposure did not reveal any significant association with cryptorchidism, as illustrated in Figure 5.

**Figure 6:**
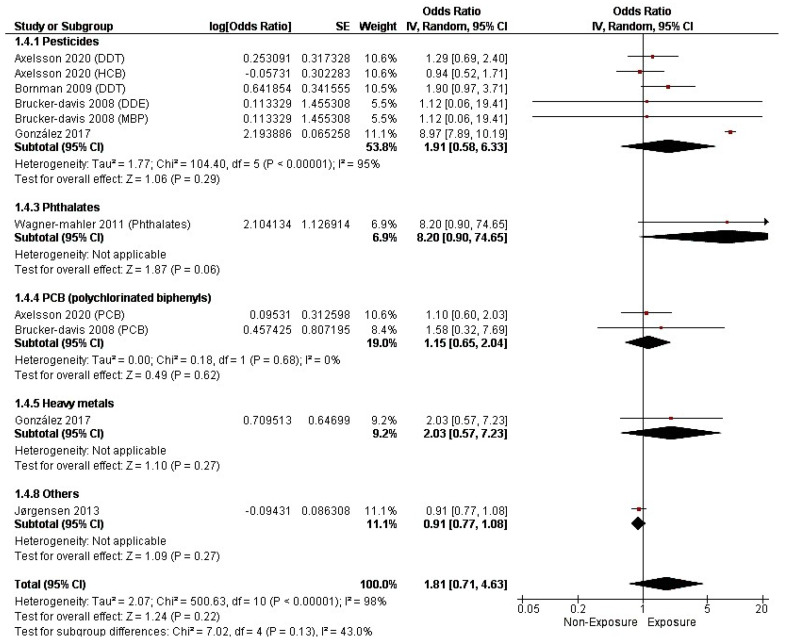
Forest plot of cryptorchidism incidence (crude OR).

The pooled cOR revealed no significant association between maternal EDC exposure and cryptorchidism (aOR = 1.81, 95% CI (0.71, 4.63), *p* = 0.22). The pooled studies demonstrated a considerable heterogeneity (I^2^ = 98%, *p* < 0.0001), as illustrated in Figure 6.

Additionally, pesticide (cOR = 1.91, 95% CI (0.58, 6.33), *p* = 0.29), phthalate (cOR = 8.20, 95% CI (0.90, 74.65), *p* = 0.06), PCB (cOR = 1.15, 95% CI (0.65, 2.04), *p* = 0.62), and heavy metal (cOR = 2.03, 95% CI (0.57, 7.23), *p* = 0.27) exposure did not reveal any significant association with cryptorchidism, as illustrated in Figure 6.

**Figure 7:**
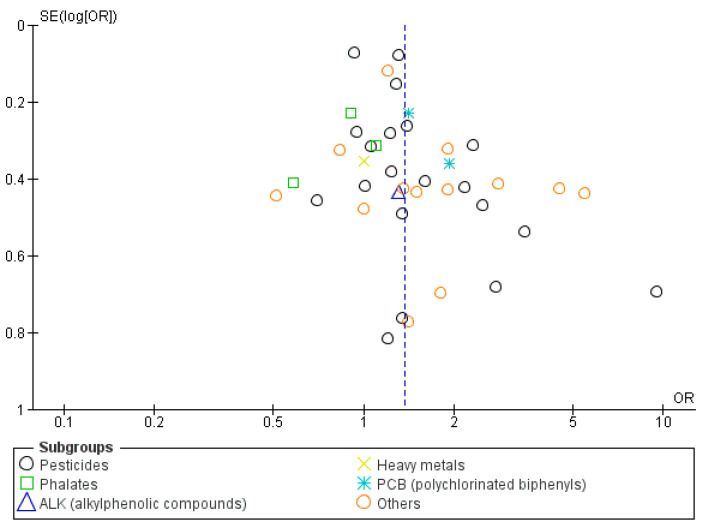
Funnel plot of cryptorchidism incidence (Publication bias).

A funnel plot based on the adjusted odds ratio was constructed to evaluate the publication bias; upon inspection, there was asymmetry, which indicates a potential publication bias, as illustrated in Figure 7.

## 4. Discussion

Maternal exposure to endocrine-disrupting chemicals (EDCs) is associated with increased risks of hypospadias and cryptorchidism. Our meta-analysis found that EDC exposure correlated with both conditions’ elevated odds ratios (aOR). Specifically, pesticides, phthalates, ALKs, and heavy metals increased the risk of hypospadias, while PCBs did not. For cryptorchidism, pesticides and PCBs were linked to elevated risk, but phthalates, ALKs, and heavy metals were not associated. The disruption of reproductive health and embryonic development due to EDC exposure may contribute to these urogenital birth defects. These EDCs include pesticides, phthalates, heavy metals, and PCBs.

Over the past few decades, social awareness regarding EDCs has significantly evolved. Initially, these compounds received limited attention, but growing scientific evidence and public concern have propelled them into the spotlight. Awareness campaigns, media coverage, and advocacy efforts have raised consciousness about the potential health risks associated with EDC exposure. As a result, regulatory bodies and policymakers now prioritize EDC research and mitigation strategies. Exposure patterns vary across different regions and countries. In the USA, extensive research on EDCs has led to regulatory actions such as the Toxic Substances Control Act (TSCA) amendments. However, challenges persist due to the vast array of chemicals in use. European countries have adopted stringent regulations through REACH (Registration, Evaluation, Authorization, and Restriction of Chemicals) and other directives. These efforts emphasize risk assessment, substitution, and transparency. Rapid industrialization and urbanization in India contribute to EDC exposure, necessitating targeted interventions to balance economic growth with environmental protection. Globally, regulatory agencies increasingly recognize the importance of EDCs. Initiatives like the Stockholm Convention address persistent organic pollutants, including some EDCs. Industry practices are shifting toward safer alternatives driven by consumer demand and regulatory pressure. Collaborative efforts between researchers, policymakers, and advocacy groups are crucial for shaping effective regulations safeguarding human health and the environment.

Our results showed that maternal EDC exposure was associated with an increased risk of hypospadias based on pooling the aOR. Specifically, pesticide exposure demonstrated a significant association with hypospadias. Das et al. [39] conducted a case-control study with 73 hypospadias cases and 146 controls. They assessed the relationship between different EDC compounds and hypospadias. Their study recorded occupational exposure to potential EDCs of 30.1% among mothers. Most of those mothers also worked as farmers or beauticians. Their findings were aligned with our study, as they concluded that maternal exposure to pesticides showed a significant association with hypospadias after adjusting for potential confounding variables. However, this study was limited by its small sample size and dependence on a job-exposure matrix to estimate the exposure, which could not confirm the actual exposure. Winston et al. [73] conducted a population-based case-control study utilizing National Birth Defects Prevention Study data. Using a US geological survey model to assess atrazine exposure, they evaluated the association between atrazine, a widely-known pesticide, and hypospadias incidence. This survey enabled the estimation of atrazine concentrations in the public water supplies and private wells based on the maternal residential address during the pivotal gestational period from weeks 6 to 16, complemented by self-reported maternal water consumption. Based on maternal water consumption, they found that maternal exposure to atrazine in Texas was significantly associated with an elevated risk of hypospadias after adjusting for the confounding variables compared to other states that had no such significant association. This could be attributed to the high concentration of atrazine in Texas compared to the other states.

Phthalates also are well-identified as potential endocrine disruptors [19]. Our study revealed that maternal phthalates exposure was correlated to an increased risk of hypospadias. Das et al. [39] findings were aligned with our results reporting the association between maternal phthalates exposure and increased risk of hypospadias. In contrast, Nassar et al. [57] reported a lack of significant association between phthalate exposure and the risk of hypospadias depending on their registry-based case-control study in Australia (1202 cases and 2583 controls). However, this study was limited by incomplete information on occupational exposure, such as the timing and the frequency of exposure, which could eventually affect the association. Our findings also showed that maternal ALK compound exposure had a significant association with hypospadias, which agrees with Das et al.’s findings [39]. However, Suarez-Varela et al. [66] found a non-significant association between ALK exposure and hypospadias after adjusting for the confounding variables based on their prospective cohort study using the Danish national base cohort between 1997 and 2009. This can be attributed to its low sample size, as it only included 262 hypospadias cases.

Nevertheless, maternal heavy metal exposure was significantly associated with hypospadias after adjustment for the potential confounders in our pooled analysis. A previous case-control study in the USA utilizing the Texas Birth Defect Registry also showed a notable association between different types of heavy metals and hypospadias [72]. According to this study, arsenic, chromium, lead, manganese, and mercury significantly correlated with hypospadias. However, cadmium and nickel did not show any significant association. Whereas Nassar et al. [57] also reported a significant association between heavy metals and hypospadias, Das et al. [39] and Suarez-Varela et al. [66] did not find a significant association between heavy metal exposure and hypospadias. It is to be mentioned that heavy metals could pass the placental barrier from mother to fetus, eventually exerting an endocrine-disrupting effect that may result in severe urogenital defects such as hypospadias, infertility, and cryptorchidism [72]. In contrast to the previously mentioned endocrine disruptors, maternal PCB exposure did not reveal any significant correlation with hypospadias. This finding agrees with that of McGlynn et al. [55], as they examined the relationship between PCB exposure and hypospadias and concluded that none of the individual PCBs investigated had a notable association with hypospadias incidence. However, the sum of PCBs showed a significant association with hypospadias. Nonetheless, this study was limited by the fact that maternal serum samples used for assessing PCB exposure were collected in the 1960s, when PCB exposure was notably greater than it is now.

Based on our pooled adjusted analysis, cryptorchidism was significantly associated with maternal EDC exposure. Pesticides, one of the most well-known EDCs, were associated considerably with cryptorchidism after adjusting for confounding variables. However, a previous study found that the boys of maternal horticultural workers had no significant association with cryptorchidism. This crucial finding could be attributed to the fact that in Denmark, pregnant women are advised to take paid sick leave or modify their work tasks when there are concerns about hazardous exposures at work [45,76]. This practice is particularly more diffuse among horticultural workers than farmers [76]. However, Pierik et al. [61] found no significant relationship between maternal exposure to HCB, HCE, and B-HCCH and cryptorchidism risk based on maternal serum levels of the three compounds in their observational study. This finding could be attributed to the low sample size (219 cases) and the low levels of these substances in mothers’ serum compared to previous studies [54,77].

Interestingly, maternal PCB exposure was associated with an elevated risk of cryptorchidism after adjustment for the confounding variables. Our primary analysis of this chemical included only two case-control studies, which found a non-significant association between PCBs and cryptorchidism [54,55], which can be regarded as a small sample size. Our crude analysis showed nearly a non-significant association between EDCs and their subgroup chemicals compared to the adjusted analysis in both outcomes. This finding shed light on the adjusted analysis’s importance and role in giving us a more accurate picture of the association between the different EDCs and urogenital defects.

To our most updated knowledge, our study is the first systematic review and meta-analysis to evaluate all the available EDCs and their association with urogenital defects. A previously published meta-analysis by Rocheleau et al. [23] investigated the association between hypospadias and pesticide exposure and included nine studies as a final number. They concluded that there is a significant association between maternal exposure to pesticides and hypospadias. Our study agreed with these findings, with more studies and more chemicals being studied. Another recently published meta-analysis assessing the relationship between maternal phthalate exposure and urogenital defects [24] was based on three studies and reported a non-significant association between maternal phthalate exposure and hypospadias or cryptorchidism. Our analysis included more studies (six in hypospadias and three in cryptorchidism) to conclude a significant association between phthalate exposure and hypospadias but a non-significant one regarding cryptorchidism. Moreover, while no drastic paradigm shifts were observed, subtle changes suggest evolving research priorities over time. The consistent inclusion of new compounds indicates ongoing scientific exploration, although the overall landscape remains relatively stable. Future investigations should continue monitoring these trends and consider the impact of evolving methodologies on our understanding of these chemical classes.

The current study provides the most updated and comprehensive evidence regarding the association between EDCs and urogenital defects. Nearly all included studies showed good and moderate quality, except for two poor-quality studies. In addition, the huge number of cases of either hypospadias (36,020 cases) or cryptorchidism (27,960 cases), as well as the geographic and ethnic variation, eventually support the generalizability of the observed results and their applicability in the real world.

However, the study also has some limitations. One of these limitations is the significant heterogeneity observed among the included studies, which can be attributed to the variation in exposure assessment methods. Whereas some studies used questionnaires, surveys, and interviews, others used serum, urine, colostrum, or cord samples to assess EDC exposure. Moreover, while our study comprehensively covers available synthetic chemicals and endocrine disruptors relevant to maternal exposure, we acknowledge that non-synthetic causes, which have historical significance dating back to Greek antiquity, were not specifically addressed. Future research should explore these non-synthetic factors to provide a more holistic understanding of urogenital defects. Also, the different concentrations of chemicals studied could be identified as another cause for this heterogeneity. A further limitation is the type of studies utilized for the analysis, as nearly all the included studies were observational, which carries a high risk for potential selection bias. In addition, some included studies were conducted on old registries, as the exposure to EDCs at the time of writing these registries was higher than now. Hence, their inclusion in our study could affect the pooled effect sizes and give us wrong estimations.

The results of the current meta-analysis underscore the crucial role of environmental and occupational exposure to EDCs as strong risk factors for developmental male reproductive system anomalies like hypospadias and cryptorchidism. To effectively manage potential risks, physicians should consider these environmental factors during prenatal consultations. Moreover, healthcare professionals should offer preventive measures for pregnant women, especially those in high-risk occupations such as farming or industrial settings where EDC exposure is common. These measures encompass implementing personal protective equipment, work setting changes, or even job adjustments throughout pregnancy. Additionally, the notable association observed in our pooled analysis advocates for the potential modifications in the prenatal screening protocols to encompass questions concerning possible EDC exposure. This would enable us to detect high-risk pregnancies early, necessitating additional monitoring or interventions.

This meta-analysis sheds light on many areas that future researchers should focus on. First, longitudinal prospective cohort studies initiated from pre-conception or early pregnancy should be conducted to aid in identifying the timing and the pivotal windows of exposure that significantly impact fetal urogenital development. Second, there is a crucial need for studies with robust exposure assessment strategies that go beyond self-reported information to involve biological markers of exposure. This approach would improve the accuracy of exposure evaluation and strengthen the causal association between EDCs and urogenital defects. Lastly, considering the profound public health implications, future research should focus more on developing and validating effective interventions to reduce EDC exposure among vulnerable populations.

## 5. Conclusions

Our pooled analysis demonstrates a significant association between maternal EDC exposure and hypospadias and cryptorchidism in male offspring after adjusting for the potential confounders. Pesticide exposure was especially associated with increased risks of both developmental anomalies. However, it is essential to interpret these results cautiously due to the heterogeneity within the methodologies of the included studies. Based on the adjusted odds ratio, phthalates, heavy metals, and ALKs revealed an increased risk of hypospadias but not cryptorchidism.

## Figures and Tables

**Table 1:** Summary and baseline characteristics of the included studies.

Study ID	Year of Publication	Study Design	Location	Number of Hypospadias Cases	Number of Cryptorchidism Cases	Number of Controls	Exposure Assessment	Maternal Characteristics			Main Chemicals	Conclusions
								Age, Mean Cases/Control (in Years)	Previous Pregnancy, N Cases/Control	Smoking, N Cases/Control		
Garcifa-Martin [45]	1996	Case-control	Spain	131	NA	243	Geographical destination-based	NA	NA	NA	Pesticides	“Our results are compatible with a hypothetical association between exposure to hormone-disruptive chemicals and the induction of cryptorchidism.”
Weidner [71]	1998	Case-control	Denmark	1345	6177	23,273	Occupation-based	NA	NA	NA	NR	“The increased risk of cryptorchidism among sons of female gardeners could suggest an association with prenatal exposure to occupationally related chemicals.”
Bhatia [30]	2004	Case-control	USA	66	75	283	Serum sample-based	27/26.6	NA	59/117	DDT	“This study does not support an association of DDT or DDE and hypospadias or cryptorchidism.”
											DDE	
Pierik [60]	2004	Case-control	The Netherlands	56	78	313	Survey-based	NA	NA	51/98	EDC	“This study suggests that paternal environmental exposures may increase the risk of cryptorchidism and hypospadias in newborn boys, which may indicate an effect on the paternal germline.”
											Pesticides	
											Solvents	
Brouwers [33]	2006	Case-control	The Netherlands	583	NA	251	Survey-based	NA	35/6	133/35	NR	“The associations found in this study support the hypothesis that genetic predisposition, placental insufficiency, and substances that interfere with natural hormones play a role in the etiology of hypospadias.”
Meyer [56]	2006	Case-control	USA	354	NA	727	Geographical destination-based	25.2/24.7	NA	NA	EDC	“Except for diclofop-methyl, we did not find evidence that estimated exposure to pesticides known to have reproductive, developmental, or endocrine disrupting effects increases the risk of hypospadias. Further research on the potential effects of exposure to diclofop-methyl is recommended.”
											Atrazine	
											Alachlor	
											Bifenthrin	
											Bromoxynil	
											Carbaryl Dicamba	
Carbone [35]	2007	Case-control	Italy	43	48	203	Survey-based	NA	NA	12-Jul	Pesticides	“The study provides only limited support to the hypothesis of a possible association between the risk of cryptorchidism and hypospadias and the occupational exposure to EDC and agricultural work.”
Fernandez [41]	2007	Case-control	Spain	50		114	Placenta tissue sample	NA	4-Jun	Nov-35	DDT	“We found an increased risk for male urogenital malformations related to the combined effect of environmental estrogens in the placenta.”
											Endosulfan I-Lindane-Mirex	
Pierik [61]	2007	Case-control	USA	NA	219	564	Serum sample-based	24/22	NA	NA	HCB	“These results provide little support for an association of cryptorchidism with exposure to low levels of HCE or HCB. For b-HCCH, the findings were somewhat suggestive of an association but inconclusive.”
											Heptachlor epoxide-b-Hexachlorocyclohexane	
Brucker-davis [34]	2008	Case-control	France	NA	78	86	Colostrum sample-based	30/30	41/44	NA	PCB	“Our results support an association between congenital cryptorchidism and fetal exposure to PCBs and possibly DDE. Higher concentrations in milk could be a marker of higher exposure or for an impaired detoxification pattern in genetically predisposed individuals.”
											DDE-mBP	
Bornman [31]	2009	Cross-sectional	Mexico	171	70	216	Survey-based	25/25	NA	NA	DDT	“Maternal exposure to DDT by living in a DDT-sprayed village was associated with having male offspring with one or more UGBDs.”
Dugas [40]	2009	Case-control	England	471	NA	490	Survey-based	NA	NA	NA	Biocide	“The authors found an association between the use of insect repellent and total biocide score and risk of hypospadias. In particular, the use of insect repellent warrants further investigation, specifically in relation to type, content, and frequency of use since this information was missing in the current study.”
											Naphthalene	
											Insect repellent	
Mcglynn [55]	2009	Case-control	USA	201	230	593	Serum sample-based	NA	NA	NA	PCB	“Given the large number of associations examined, these findings do not strongly support the hypothesis that PCBs are associated with cryptorchidism or hypospadias. Because population serum PCB levels at the time of sample collection were considerably higher than at present, it is unlikely that current PCB exposure is related to the development of either anomaly.”
Nassar [57]	2009	Case-control	Australia	1202	NA	2583	Survey-based	28.2/27.9	NA	NA	EDC	“Our findings provide preliminary evidence of an association between exposure to EDCs with oestrogenic or antiandrogenic properties and increased risk of hypospadias.”
											Pesticides-POC-ALK-BPC-Heavy metals-Phthalates.	
Ormond [58]	2009	Case-control	England	471	NA	490	Survey-based	NA	NA	113/88	Hair spray	“Excess risks of hypospadias associated with occupational exposures to phthalates and hair spray suggest that antiandrogenic EDCs may play a role in hypospadias. Folate supplementation in early pregnancy may be protective.”
											Cleaning agents	
											Printing ink	
											Exhaust fumes	
Palmer [59]	2009	Cohort	USA	NA	38	NA	Survey-based	NA	NA	NA	diethylstilbestrol	“These results indicate that prenatal exposure to DES increases the risk of male urogenital abnormalities and that the association is strongest for exposure that occurs early in gestation. The findings support the hypothesis that endocrine disrupting chemicals may cause the increased prevalence of cryptorchidism that has been seen in recent years.”
Carmichael [36]	2010	Case-control	USA	20	NA	28	Serum sample-based	NA	NA	NA	PCB	“Levels of the PBDEs and PCBs were not statistically significantly different, but the sample size was small. The current study adds to a relatively limited knowledge base regarding the potential association of specific contaminants with hypospadias or other birth defects.”
											PBDE	
Giordano [46]	2010	Case-control	Italy	80	NA	80	Serum sample-based	NA	NA	NA	EDC	“This study, although based on a limited number of cases, for the first time provides evidence of an association between maternal exposure to EDCs, in particular elevated plasma hexachlorobenzene concentration, and the development of hypospadias in the offspring.”
											Polychlorinated organic compounds	
											ALK	
											Biphenolic compounds	
											Heavy metals	
Gabel [44]	2011	Cohort	Denmark	11	17	477	Survey-based	NA	NA	NA	NR	“The data are compatible with a slightly increased risk of cryptorchidism in sons of women exposed to pesticides by working in horticulture.”
Rocheleau [74]	2011	Case-control	USA	647	NA	1496	Survey-based	NA	NA	NA	Insecticides	“Using broad classes of insecticides, herbicides, and fungicides, we found no evidence that low intensity maternal periconceptional occupational pesticide exposure was a risk factor for hypospadias.”
											Herbicides	
											Fungicides	
Shekharyadav [64]	2011	Case-control	India	80	NA	120	Survey-based	NA	NA	NA	EDC	“Our study suggests irrespective of genetic predisposition, higher level of some OCPs may be associated with increased risk of hypospadias.”
Suarez-Varela [66]	2011	Cohort	Spain	262	1002	NA	Survey-based	NA	NA	NA	EDC	“The study provides some but limited evidence that occupational exposure to possible endocrine disrupting chemicals during pregnancy increases the risk of hypospadias.”
											Pesticides	
											Organochlorine	
											compounds	
											Phthalate esters	
											ALK	
											Heavy metals	
											Bis-phenols	
Trabert [67]	2011	Case-control	USA	197	217	557	Serum sample-based	24/22	NA	210/250	trans-Nonachlor-Oxychlordane	“The results do not support an association between chlordane levels and cryptorchidism or hypospadias. It is unlikely that current chlordane exposure is related to the development of either anomaly, given that serum chlordane levels at the time of sample collection, the early 1960s, were considerably higher than levels at present.”
Wagner-mahler [69]	2011	Case-control	France	NA	95	188	Survey-based	NA	NA	NA	Phthalates	“Our results suggest that maternal exposure to anti-rust or phthalates could be a risk factor, whereas eating fruits daily seemed somewhat protective. The prevalence of cryptorchidism in our area is on the lower bracket compared with other countries and is associated with familial and environmental risk factors.”
											Heavy metals	
Rignell-hydbom [62]	2012	Case-control	Sweden	237	NA	237	Serum sample-based	NA	NA	NA	PCB	“The present study suggests that fetal exposure to HCB and p,p’-DDE may be a risk factor for hypospadias.”
											DDE	
											HCB	
Agopian [28]	2013	Case-control	USA	8909	4324	16,433	Survey-based	NA	NA	1025/989	Atrazine	“In summary, we report on modest, consistent, inverted U-shaped associations between estimated maternal residential exposure to atrazine and several genital malformations in male offspring.”
Carmichael [37]	2013	Case-control	USA	690	NA	2195	Survey-based	NA	382/1411	NA	Insect growth regulator	“Most pesticides were not associated with elevated hypospadias risk. For the associated few, results should be interpreted with caution until replicated in other study populations.”
											Halogenated organic	
											monochlorophenoxy	
											acid or ester	
											Organochlorine-Organotin	
											Phosphine-Thiocarbamate	
Jorgensen [51]	2013	Cohort	Denmark	33	134	NA	Occupation-based	NA	NA	NA	NR	“Our nationwide cohort study shows that, despite exposure to a complex chemical milieu, hairdressers do not have an increased risk of having boys with cryptorchidism and hypospadias.”
Vesterholmjensen [68]	2013	Case-control	Denmark-Finland	NA	215	108	Cord sample-based	NA	NA	NA	PFOA	“Our data indicate that women in Denmark and Finland are generally exposed to PFOA and PFOS, but there are differences in exposure levels between countries. We found no statistically significant association between cord blood PFOA and PFOS levels and congenital cryptorchidism; however, our study was small, and larger studies are warranted.”
											PFOS	
Jorgensen [52]	2014	Cohort	Denmark	NA	229	NA	Occupation-based	NA	NA	NA	NR	“This nationwide cohort study found a slightly increased risk of cryptorchidism in sons of maternal horticultural workers and farmers. However, subgroup analyses indicated similar findings for paternal horticultural workers and no association for women likely working in the first trimester. The main findings should, therefore, be interpreted with caution.”
Fernandez [42]	2015	Case-control	Spain	28		51	Placenta tissue sample	29/30	16-Apr	17-May	Methyl-PB-ethyl-PB	“The multivariable regression analyses indicated a statistically significant association between exposure to BPA and propyl-PB and the risk of malformations.”
											Propyl-PB-butyl-PB	
Jensen [50]	2015	Case-control	Denmark	75	270	300	Amniotic fluid sample	NA	NA	NA	Phthalate	“Data on the DEHP metabolite indicate possible interference with human male fetal gonadal function. Considering the DiNP metabolite, we cannot exclude (nor statistically confirm) an association with hypospadias and, less strongly, with cryptorchidism.”
Kalfa [53]	2015	Case-control	France	300	NA	302	Survey-based	NA	NA	NA	EDC	“Our multi-institutional study showed that parental professional, occupational, and environmental exposures to chemical products increase the risk of hypospadias in children.”
											Pesticides	
											Cosmetics	
											Herbicides	
											Detergents	
											ALK	
											Phthalates	
											Heavy metals	
Koskenniemi [54]	2015	Case-control	Turkey-Denmark-Finland	NA	44	38	Serum sample-based	NA	NA	NA	PCB	“Prenatal exposure to PCDD/Fs and PCDD/F-like PCBs may be associated with increased risk for cryptorchidism. Our finding does not exclude the possibility of an association between the exposure to PBDEs and cryptorchidism.”
											PBDE	
Haraux [48]	2016	Case-control	France	57	NA	162	Survey-based	29.7/28.7	NA	NA	EDC	“Our results suggest that maternal occupational exposure to EDCs is a risk factor for hypospadias and suggests a possible influence of household use of hair cosmetics during early pregnancy on the incidence of hypospadias in the offspring.”
											Hair cosmetic	
											Insecticides	
Winston [73]	2016	Case-control	USA	343	NA	1422	Geographical destination-based	NA	NA	NA	Atrazine	“While the association that we observed was weak, our results suggest that additional research into a possible association between atrazine and hypospadias occurrence, using a more sensitive exposure metric, would be useful.”
Gonzalez 2017 [47]	2017	Case-control	Spain	678	963	587,142	Geographical destination-based	NA	NA	NA	Pesticides	“Data on environmental exposure to pesticides and gestational disorders are scarce. A population-based case-control study estimated the risk of maternal-infant disorders. Prevalence and risk of reproductive disorders and congenital anomalies were estimated. Higher prevalences and risk were observed in areas of high exposure to pesticides. Environmental pesticides can be risk factors for developing maternal-infant disorder.”
Warembourg [70]	2017	Case-control	France	15	14	86	Urine sample-based	NA	NA	19/9	MAA	“In view of the toxicological plausibility of our results, this study, despite its small sample size, raises concern about the potential developmental toxicity of MAA on the male genital system and calls for thorough identification of current sources of exposure to MAA.”
											PhAA	
Haraux [49]	2018	Case-control	France	25	NA	58	Meconium sample-based	29/28.2	NA	21-Sep	Diazinon	“We conclude that prenatal exposure to these two herbicides (as assessed by meconium analysis) correlated with isolated hypospadias. The results of our case-control study (i) suggest that prenatal exposure to pesticides interferes with the development of the male genitalia, and (ii) emphasize the importance of preventing pregnant women from being exposed to EDCs in general and pesticides in particular.”
											Malathion	
											DETP	
											DEP	
											DMP	
											Isoproturon	
											Desmethylisoproturon	
											MCPA	
Cognez [38]	2019	Case-control	France	50	123	8199	Survey-based	NA	NA	30/1679	Pesticides	“Our results suggest a possible increased risk of hypospadias associated with prenatal use of some domestic pesticide products, likely to contain insecticides, and of cryptorchidism with nearby orchard acreage (crops repeatedly sprayed with pesticides). This work is limited by its modest number of cases.”
Estors Sastre [75]	2019	Case-control	Spain	210		210	Survey-based	NA	NA	33/29	EDC	“Advanced age, some parental occupational exposure to EDCs, some drug consumption, smoking, and the father’s history of urological disorders may increase risk and predict the developments of these malformations. Studies with larger sample sizes are needed to assess associations between individual EDC occupational exposures and drugs and these malformations.”
White [72]	2019	Case-control	USA	8981	NA	89,806	Geographical destination-based	NA	NA	576/5280	Heavy metals	“Using data from one of the world’s largest active surveillance birth defects registries, we identified significant associations between hypospadias and HMHAP exposures.”
Axelsson [29]	2020	Case-control	Sweden	NA	165	165	Serum sample-based	29/28	NA	15/16	PCB	“We found no evidence of an association between maternal levels of PCB or HCB during the pregnancy and the risk of having cryptorchidism in the sons.”
											DDE	
											HCB	
Fisher [43]	2020	Case-control	UK	NA	30	275	Serum sample-based	32.98/33.54	NA	0/4	Phthalates	“Our observational findings support experimental evidence that intrauterine exposure to BPA and n-PrP during early gestation may adversely affect male reproductive development. More evidence is required before specific public health recommendations can be made.”
											BPA	
											TCS	
											BP-3	
Bougneres [32]	2021	Case-control	France	8766	13,105	43,830	Geographical destination-based	NA	NA	NA	NR	“Our study supports that children born to mothers living close to a vineyard have a two-fold increased risk of H. For environmental research, using VC = provides an alternative to a classical case-control technique.”
Spinder [65]	2021	Case-control	The Netherlands	364	NA	5602	Survey-based	NA	NA	NA	EDC	“Women, their healthcare providers, and their employers need to be aware that occupational exposure to specific EDCs early in pregnancy may be associated with CAKUT in their offspring. An occupational hygienist should be consulted in order to take exposure to those specific EDCs into consideration when risk assessments are carried out at the workplace.”
											Pesticides	
											ALK	
											Phthalates	
											Benzophenones	
											parabens-siloxanes	
Das [39]	2023	Case-control	India	37	NA	146	Survey-based	NA	NA	NA	Pesticides	“This study suggests that EDCs are a risk factor for hypospadias through occupational exposure during fetal life.”
											Phthalates	
											ALK	
											Heavy metals	
Rouget [63]	2024	Case-control	France	69	NA	135	Meconium sample-based	NA	35/39	21/36	Nitrophenol-diethyl phosphate-Fenitrothion-Carbofuran	“Our small study provides a robust assessment of fetal exposure. Fenitrothion’s established antiandrogenic activities provide biological plausibility for our observations. Further studies are needed to confirm this hypothesis.”

Abbreviations: NA = Not applicable; NR = Not Reported; DDT = dichlorodiphenyl trichloroethane; EDC = Endocrine-disruptors chemicals; HCB = Hexachlorobenzene; PCB = polychlorinated biphenyls; ALK = alkylphenolic compounds; BPA = bisphenol; DETP = diethyl phosphate; DDE = dichloro diphenyl dichloro ethylene; PHAA = phenoxy acetic acid; MAA = methoxy acetic acid; DES = diethylstilbestrol; PFOS = perfluorooctanesulfonic acid; PFOA = perfluorooctanoic acid; BP-3 = Benzophenone-3; TCS = triclosan; OR = Odds ratio.

## Data Availability

All data used in this systematic review and meta-analysis are included in this article and its Supplementary Materials or are publicly available from the original sources.

## Supplementary Materials

The following supporting information can be downloaded at: https://www.mdpi.com/article/10.3390/metabo14090477/s1, Supplementary Table S1: A detailed search strategy for each database is outlined; Supplementary Table S2: Quality assessment of the included case-control studies by NOS tool; Supplementary Table S3: Quality assessment of the included cohort studies by NOS tool.
