# Supplementary material for: The Association between Maternal Endocrine-Disrupting Chemical Exposure during Pregnancy and the Incidence of Male Urogenital Defects: A Systematic Review and Meta-Analysis
Source: Metabolites. 2024 Aug 29;14(9):477. doi: 10.3390/metabo14090477 (PMC11434617; doi:10.3390/metabo14090477)
Supplement: Supplementary file 1 [file metabolites-14-00477-s001.zip › Supplmentary table S1.pdf]

**Supplementary Table 1:** databases were used to search for articles related to the following key words:		
**Databases**	**Search Strategy**	**Results**
Pubmed	("Endocrine disruptors" OR "Endocrine disrupting chemical" OR "Endocrine disrupting chemicals" OR EDC OR DDT OR dichlorodiphenyldichloroethylene OR DDE OR Pyrethroid OR Pyrethroids OR Pesticides OR Pesticide OR phthalates OR phthalate OR "heavy metal" OR hexachlorobenzene OR "Polychlorinated compounds" OR Alkylphenol OR Bisphenol OR perfluoroalkyl OR fluorocarbons OR fluorocarbon OR Triclosan) AND ("Urogenital defect" OR "Urogenital malformation" OR "Genital defect" OR "Genital malformation" OR Hypospadias OR cryptorchidism)	489
Scopus	TITLE-ABS-KEY (("endocrine disruptors" OR "endocrine disrupting chemical" OR "endocrine disrupting chemicals" OR edc OR ddt OR dichlorodiphenyldichloroethylene OR dde OR pyrethroid OR pyrethroids OR pesticides O R pesticide OR phthalates OR phthalate OR "heavy metal" OR hexachlorobenzene OR "polychlorinated compounds" OR alkylphenol OR bisphenol OR perfluoroalkyl OR fluorocarbons OR fluorocarbon OR triclosan) AND (" urogenital defect" OR "urogenital malformation" OR "genital defect" OR "genital malformation" OR hypospadias OR cryptorchidism))	818
Web of science	(ALL=(("Endocrine disrupting chemical" OR "Endocrine disrupting chemicals" OR EDC OR DDT OR dichlorodiphenyldichlorothylene OR DDE OR Pyrethroid OR Pyrethroids OR Pesticides OR Pesticide OR phthalates OR phthalate OR "heavy metal" OR hexachlorobenzene OR "Polychlorinated compounds" OR Alkylphenol OR Bisphenol OR perfluoroalkyl OR fluorocarbons OR fluorocarbon OR Triclosan))) AND ALL=(("Urogenital defect" OR "Urogenital malformation" OR "Genital defect" OR "Genital malformation" OR Hypospadias OR cryptorchidism))	565
Cochrane library	("Endocrine disruptors" OR "Endocrine disrupting chemical" OR "Endocrine disrupting chemicals" OR EDC OR DDT OR dichlorodiphenyldichloroethylene OR DDE OR Pyrethroid OR Pyrethroids OR Pesticides OR Pesticide OR phthalates OR phthalate OR "heavy metal" OR hexachlorobenzene OR "Polychlorinated compounds" OR Alkylphenol OR Bisphenol OR perfluoroalkyl OR fluorocarbons OR fluorocarbon OR Triclosan) AND ("Urogenital defect" OR "Urogenital malformation" OR "Genital defect" OR "Genital malformation" OR Hypospadias OR cryptorchidism) in Title Abstract Keyword - (Word variations have been searched)	11

The total from the four databases:		1883
Number of duplicates:		600
Number after removing duplication:<br>(By Endnote):		1283
