# Supplementary material for: The Association between Maternal Endocrine-Disrupting Chemical Exposure during Pregnancy and the Incidence of Male Urogenital Defects: A Systematic Review and Meta-Analysis
Source: Metabolites. 2024 Aug 29;14(9):477. doi: 10.3390/metabo14090477 (PMC11434617; doi:10.3390/metabo14090477)
Supplement: Supplementary file 1 [file metabolites-14-00477-s001.zip › Supplmentary table S3.pdf]

Study ID	Supplementary Table 3: Quality assessment of the included cohort studies by NOS tool								
	Selection				Comparability	Outcome			Quality Score
	Representativeness of the exposed cohort	Selection of the nonexposed cohort	Ascertainment of exposure	Demonstration that outcome of interest was not present at the start of the study	Comparability of cohorts on the basis of the design or analysis	Assessment of outcome	Was follow-up long enough for outcomes to occur	Adequacy of follow-up of cohorts	
**Jørgensen 2013**	*	*	*		*	*	*	*	Good
**Jørgensen 2014**	*	*	*		*	*	*	*	Good
**Palmer 2009**	*	*				*	*	*	Moderate
**Suárez-Varela 2011**	*	*	*		*	*	*	*	Good
**Wagner-mahler 2011**	*	*	*		*	*	*	*	Good
**Gabel 2011**	*	*	*		*	*	*	*	Good
